# Prediction of oxygen uptake dynamics by machine learning analysis of wearable sensors during activities of daily living

**DOI:** 10.1038/srep45738

**Published:** 2017-04-05

**Authors:** T. Beltrame, R. Amelard, A. Wong, R. L. Hughson

**Affiliations:** 1 Faculty of Applied Health Sciences, University of Waterloo, Waterloo, Ontario, Canada; 2 Conselho Nacional de Desenvolvimento Científico e Tecnológico (CNPq), Brasília, Distrito Federal, Brazil; 3 Departamento de Fisioterapia, Universidade Ibirapuera, São Paulo, Brazil; 4 Department of Systems Design Engineering, University of Waterloo, Waterloo, Ontario, Canada; 5 Schlegel-University of Waterloo Research Institute for Aging, Waterloo, Ontario, Canada

## Abstract

Currently, oxygen uptake (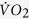) is the most precise means of investigating aerobic fitness and level of physical activity; however, 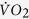 can only be directly measured in supervised conditions. With the advancement of new wearable sensor technologies and data processing approaches, it is possible to accurately infer work rate and predict 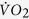 during activities of daily living (*ADL*). The main objective of this study was to develop and verify the methods required to predict and investigate the 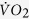 dynamics during *ADL*. The variables derived from the wearable sensors were used to create a 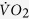 predictor based on a random forest method. The 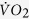 temporal dynamics were assessed by the mean normalized gain amplitude (*MNG*) obtained from frequency domain analysis. The *MNG* provides a means to assess aerobic fitness. The predicted 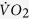 during *ADL* was strongly correlated (*r* = 0.87, *P* < 0.001) with the measured 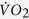 and the prediction bias was 0.2 ml·min^−1^·kg^−1^. The *MNG* calculated based on predicted 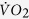 was strongly correlated (*r* = 0.71, *P* < 0.001) with *MNG* calculated based on measured 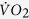 data. This new technology provides an important advance in ambulatory and continuous assessment of aerobic fitness with potential for future applications such as the early detection of deterioration of physical health.

The measurement of oxygen uptake (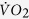) responses in steady-state condition is commonly used to precisely estimate the individual energy expenditure of a given physical activity^1^. Besides energy expenditure estimation, the evaluation of the temporal dynamics of the 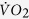 during physical activity transitions can provide valuable information about the aerobic system integrity^2^,^3^. From a practical perspective, abnormal aerobic responses to exercise may precede the clinical detection of non-communicable diseases^4^. Therefore, wearable technologies that continuously evaluate the aerobic response during non-supervised activities of daily living (*ADL*) have the potential to identify not only changes in physical fitness, but also disease states before the manifestation of clinical symptoms^5^,^6^.

In parallel with the advances in wearable devices, machine learning (*ML*) techniques are becoming popular to analyze the large quantities of longitudinal data streamed from these devices^7^. The *ML* algorithms may provide the technical basis to better identify non-trivial and complex patterns in long-term continuous biological signals^8^. The data mining process by *ML* is often based on the relationship between known inputs and outputs (supervised learning)^9^. The initial crude algorithms are feed with input and known output data (examples), and evolve according to the general structures that describe the input-output relationships. When the algorithm reaches a satisfactory generalization capacity, the output can be estimated by the inputs through a set of rules nested within the algorithm.

In this study *ML* will be used to build a 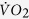 predictor based on inputs provided by wearable sensors. The main objective of this study was to predict and evaluate the temporal dynamics of the aerobic response during realistic activities. Specifically, data acquired from wearable sensors fusion will be processed by *ML* algorithms to predict the 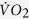 data with subsequent aerobic system analysis. The hypothesis of this study is that the signals collected by wearable sensors contain latent features that allow the characterization of the aerobic system response to exercise.

## Methods

### Study design

Sixteen healthy, active male adults enrolled in this study (27 ± 7 years old, 174 ± 7 cm and 78 ± 14 kg). A written, informed consent was obtained from all participants. The Office of Research Ethics at the University of Waterloo reviewed and approved the research procedures (ID: ORE20931) that were consistent with the Declaration of Helsinki.

**Figure 1:**
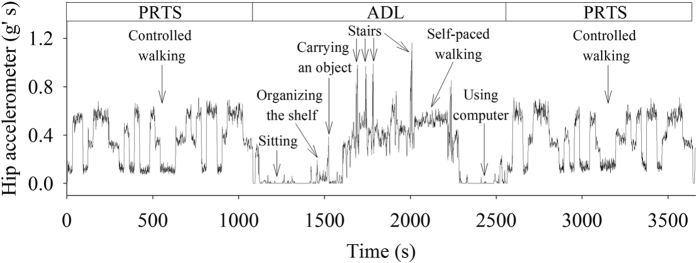
Representative hip acceleration response during pseudorandom ternary sequence (PRTS) walking protocol and simulated activities of daily living (*ADL*). The arrows point to each specific *ADL* (labels).

As opposed to previous studies^9^,^10^,^11^,^12^ that used treadmill ergometers, participants performed two pseudorandom ternary sequence (PRTS) over-ground walking protocols^13^ separated by simulated *ADL*. Considering a step duration of 30 s, the PRTS was generated according to previous literature^13^,^14^,^15^. The PRTS was composed by a warm-up period of 300 s of extra sequence followed by 13 min of protocol. The walking cadences alternated between three levels (75, 105 or 135 steps·min^−1^). These levels corresponded to ≈ ±30% of the normal walking cadence^16^. The simulated *ADL* protocol (≈20 min) was composed by sitting, organizing the shelf, carrying objects (≈4.5 Kg), stairs (four up and four down flights of stairs), self-paced walking and sitting using the computer. Figure 1 exemplifies the behaviour of the hip acceleration (further explained) during these protocols.

### Data acquisition

Throughout the PRTS and simulated ADL, the 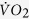 data were measured breath-by-breath by a portable metabolic system (K4b^2^, COSMED, Italy). The gas concentrations and air volume/flow were calibrated following manufacturer’s specifications before each visit. The wearable sensors hip accelerometer, ECG and respiration band were integrated into a smart shirt (Hexoskin^®^). The raw sensor signals were used to obtain heart rate (*HR*), minute ventilation (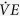), breathing frequency (*BF*), total hip acceleration (*H*_*acc*_), and walking cadence (*CAD*) through previously validated proprietary algorithms^17^. From the *HR* data, a new variable was derived. The Δ*HR* was composed by the difference between the current *HR* value and the previous value by a 1 s lag operator, capturing dynamic changes in cardiac activity. The combination of the accelerometer sample rate (64 Hz), resolution (0.004 g) and range (16 g’s) was sufficient to capture all expected *ADL* movements^18^. The data from the wearable sensors and the 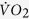 signal were synchronized, linearly interpolated, and re-sampled at 1 Hz.

### Machine learning

**Figure 2:**
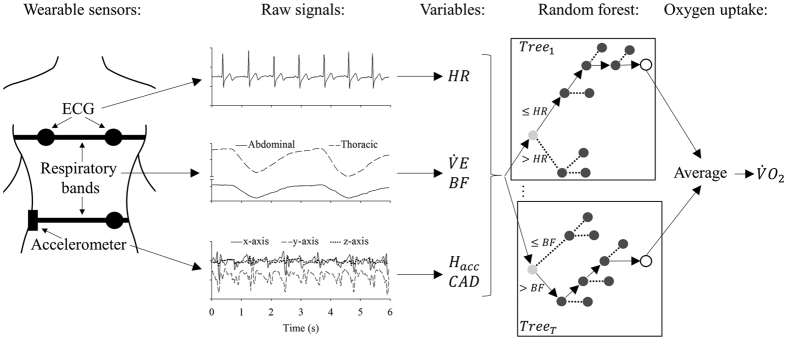
Illustration of the transformation of wearable sensor signals into oxygen uptake (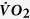) by a random forest regression model. This algorithm was created based on a machine learning approach (see text for details). The heart rate (HR) was estimated based on the ECG signal. The Δ*HR* variable consisted of the difference between the current *HR* value with the previous value. The ventilation minute (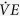) and breathing frequency (*BF*) were estimated based on two respiratory bands (abdominal and thoracic). The hip acceleration (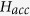) and walking cadence (*CAD*) were estimated based on tri-axis (x, y and z axis) accelerometer located at the hip. These variables were considered as inputs to a random forest algorithm consisting of a ruleset (regression trees, 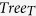) composed by thresholds that split the signal into two tree branches (light grey circles). The numerical output (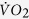) was the average across the individual trees’ leaf nodes (open circles).

As demonstrated in Fig. 2, the 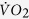 predictor was based on a random forest machine learning method^19^. The re-sampled 1 Hz data for *HR*, Δ*HR* 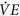, *BF, H*_*acc*_, *CAD* and 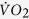 were low-pass filtered at 0.01 Hz. Because frequencies higher than 0.01 Hz can be affected by non-linearities introduced by circulatory distortions^20^, they were filtered out to diminish their potential impact on the machine learning algorithm. Data mining was performed in Matlab R2016a (MathWorks, Natick, MS, USA).

As described in Code I (Supplementary material), the tested algorithms were validated by leave-one-participant-out cross-validation^21^. This validation was chosen to avoid data overlapping between training and testing datasets which might mislead the prediction accuracy evaluation^8^. The mined algorithm accuracy was evaluated by the average of the Pearson’s linear correlation coefficient (*r*) of all folds from the validation process. The time series data and the ability of the predictor to estimate the system temporal dynamics (further explained) were considered into this validation.

Ensemble models (i.e., “super” machine learning models that combine the output of individual models within) have gained popularity for outperforming singular models with large complex data^22^. The random forest model is a popular ensemble model that does not make any inherent assumptions about data distribution (e.g., normality). A random forest model comprises a set of individual binary decision trees (see Fig. 2), which are grown using some elements of randomization. To generate a 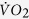 prediction given a set of measurement features, each tree evaluates its hypothesis based on the input, and the random forest model conducts a “voting” scheme where all of the individual tree predictions are aggregated to generate a final estimate, effectively reducing potential incorrect outlier estimations. Of particular interest, random forests treat the feature space as clustered disjoint sets of target (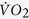) values, which is helpful for aggregating many data points that may be similar but vary due to measurement noise. When building individual trees, the method actively seeks data that improve the fit. Finally, prediction is fast, only requiring fast tree traversals. These properties make it a good candidate for real-time 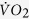 prediction for future implementations in embedded systems.

For this study, the random forest model was implemented as an ensemble of bootstrap aggregate regression trees. Specifically, each tree is made up of nodes with up to two children nodes, starting with the root node and traversing down to the end. A node contains a splitting criterion (e.g., *HR* > 50 bpm). For each time point, the feature values were evaluated by traversing the nodes to the bottom of the tree based on their decision values. Each bottom node, the “leaf node”, contains the tree’s estimated output for the given feature values. Each regression tree was grown individually with a randomly sampled subset of the training data. The final estimated 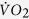 value for a given time point was computed as the average prediction across all the tree’s leaves.

Mathematically, let *X* = [x_1_, … x_*n*_] be a set of *n* feature vectors, and *Y* = [y_1_, … y_*n*_] be the (known) 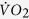 value corresponding to each feature set. The goal was to develop a random forest of *T* individual regression trees. Each individual regression tree was trained on a random data sample (in-bag selection) for generalizability. Each tree was grown node-by-node as follows. For each node, a random 1/3 subset of the features was selected as candidate splitting features. An optimal node split (into left and right subtrees) was sought such that it minimized the sum of squared residuals in the two prospective subsets:


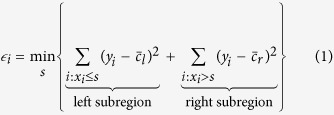


where *s* is the splitting value, *x*_*i*_ is the candidate splitting feature, *y*_*i*_, and 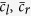 are the mean responses from the prospective left and right subregion respectively. The feature that exhibited the smallest 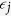 was chosen as this node’s splitting criterion:


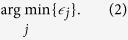


This process was repeated recursively for each node, until a full tree was grown. Thus, given a new feature vector x, each tree predicted the 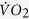 value 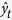 by following the binary splits according to the given feature vector and outputting the leaf node’s prediction value where a leaf node (dark grey circles in Fig. 2) is a node in the tree that doesn’t have any split (light grey circles in Fig. 2). The final predicted 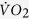 value was computed by the bag’s weighted average of the individual tree predictions:


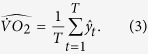


Since each random forest, one per participant, was validated individually, a total of sixteen random forest were generated at the end of the leave-one-participant-out cross-validation (Supplementary Material, Code I). Each random forest contained a set of decision trees that predicted 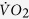 data based on an optimal split of the features. Beyond these sixteen random forests, the output from all forests were ensemble averaged to reduce system noise, resulting in a final 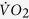 predictor.

### Oxygen uptake dynamics evaluation

The data corresponding to the PRTS protocol, optimized for system identification^13^,^15^, was used for the evaluation of the aerobic system dynamics. The *H*_*acc*_ data were considered as system inputs and the measured and predicted 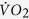 as outputs. To increase the signal-to-noise ratio, input and output responses during each of the two PRTS were time aligned and averaged to obtain a single PRTS response per participant. Fast Fourier Transformations were used to convert the data from time to frequency domain. The responses were analyzed between the fundamental frequency (0.0012 Hz) to 0.008 Hz to constrain the analysis of 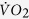 dynamics to a range where linear first-order behaviour has been documented^20^. Non-linearities might introduce misinterpretation about the aerobic system temporal dynamics study where the 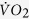 dynamics at higher frequencies might be also a consequence of circulatory distortions^20^.

As a characteristic of the PRTS protocol^15^, the amplitudes for the even harmonics were excluded a priori due to the absence of system stimulus. As previously proposed^13^,^20^,^23^, the system gains at the different frequencies (output/input ratio) were normalized by the gain at the first harmonic. This procedure eliminates the influence of the system static gain over the temporal characteristics of the system which ultimately are related to aerobic power^24^,^25^,^26^,^27^. Finally, the mean of the normalized gains (*MNG*) was used as an index of the system temporal dynamics. Higher *MNG* values mean faster aerobic responses. The algorithm for *MNG* calculation is described in the Supplementary Material (Code I).

### Statistical analysis

For each participant and considering the entire group response, the predicted 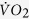 data were validated during the PRTS and *ADL* using the raw measured 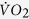 data as reference (without 0.01 Hz high pass filtering). The *MNG* estimated from the predicted 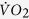 was also validated using the *MNG* estimated from the measured 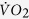 as reference. The *r* coefficient, Bland-Altman plot, confidence interval (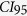) and Student t-test were used for data validation. The prediction bias (measured minus predicted) was also compared with the equality line (bias = 0) by Student t-test. To further explore the predictions during *ADL*, the sample was also clustered into three groups according to the metabolic equivalent (*METS*) estimated from the measured 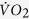 (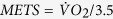). The first cluster was composed by the resting metabolic rate (*RMR*) estimated from the 60 s average of the 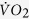 response during resting. Since the exercise protocol designed for this study was focused on realistic *ADL* that is in majority composed by light and moderate intensity^28^, less than 2% of the experimental data were composed by *METS* higher than 6.0. Therefore, the average of the samples within the intervals 2–3.9 (505 ± 137 samples per participant) and 4.0–5.9 (422 ± 67 samples per participant) were grouped as light and moderate activities, respectively^29^.

## Results

**Figure 3:**
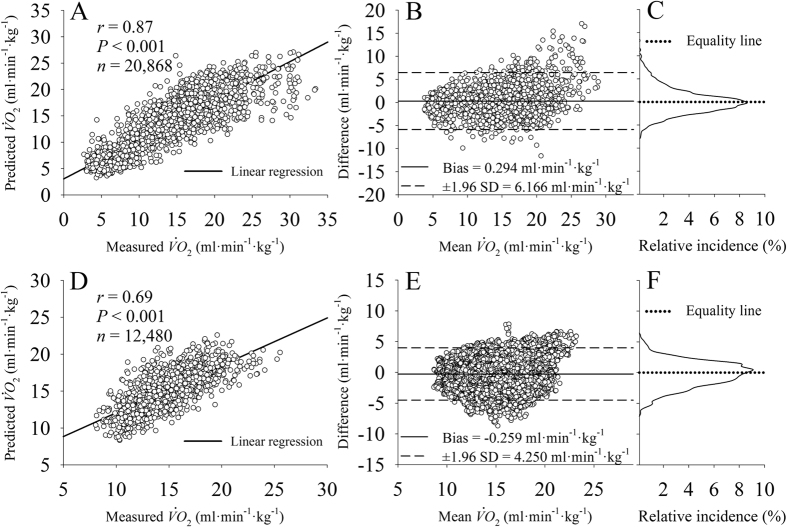
Data were down sampled to 0.1 Hz for a better data visualization. Graphs (**A**), (**B**) and (**C**) are related to data obtained during activities of daily living (~1200 samples per participant) and graphs (**D**), (**E**) and (**F**) are related to data obtained during pseudorandom walking protocol (1560 samples per participant). (**A**) and (**D**): linear correlation of the measured and predicted oxygen uptake (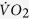) between all participants. (**B**) and (**E**): Bland-Altman plot of the predicted and measured 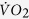 data. (**C**) and (**F**): distribution of the prediction error.

Figure 3 displays the comparison of the measured and predicted 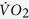. The data obtained during *ADL* are displayed in Fig. 3A–C and the data obtained during the PRTS protocol are displayed in Fig. 3D–F. As demonstrated in Fig. 3A, the quality of the prediction was verified by a strong and significant positive correlation (*r* = 0.87, *P* < 0.001 and *n* = 20,868) with the measured data during *ADL*. By individually analyzing the correlation level, all participants presented a strong and significant positive correlation (*r* = 0.88 ± 0.05, *P* < 0.001 ± 0.00 and *n* = ≈1200 per participant) between predicted and measured data with a bias of 0.331 ± 1.187 ml·min^−1^·kg^−1^. The Bland-Altman plot for the measured and predicted 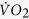 during *ADL* is shown in Fig. 3B. Considering the entire sample for *ADL*, the bias (0.294 ml·min^−1^·kg^−1^, ≈2.2% of the average response) was statistically (*P* < 0.05) higher than the equality line. The 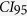 was 6.166 ml·min^−1^·kg^−1^ around the bias. The relative distribution of the error is plotted in Fig. 3C. The error distribution followed a Gaussian-like function with the majority of the error located close to the equality line (bias = 0).

Considering all data points from all participants during PRTS (Fig. 3D), the correlation coefficient was strongly positively correlated (*r* = 0.69, *P* < 0.001 and *n* = 12,480). By individually analyzing the correlation level, all participants presented a strong and significant positive correlation (*r* = 0.77 ± 0.09, *P* < 0.001 ± 0.00 and *n* = 780 per participant) between predicted and measured data. The Bland-Altman plot for the measured and predicted 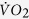 during PRTS is shown in Fig. 3E. The bias of the prediction, −0.259 ml·min^−1^·Kg^−1^ was lower (*P* < 0.001) than the equality line representing only ≈1.7% of the average response during the PRTS protocol. The 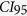 was 4.250 ml·min^−1^·kg^−1^. The relative distribution of the error is plotted in Fig. 3F. This distribution also followed a Gaussian-like function with the majority of the error located close to the equality line (bias = 0).

### Metabolic equivalent

**Figure 4:**
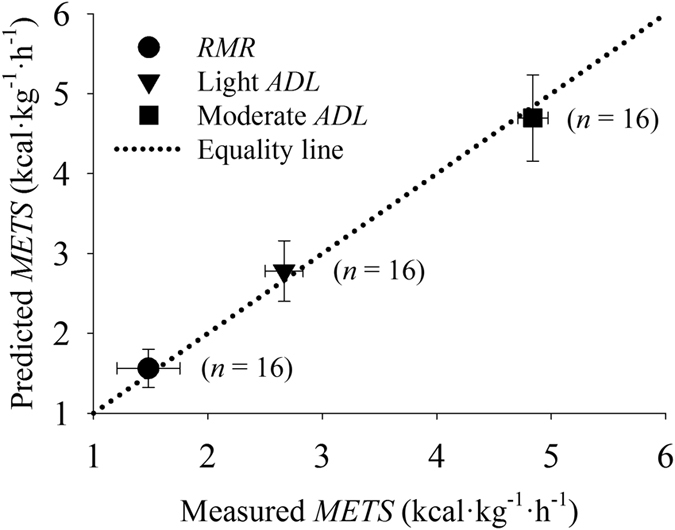
Relationship between the measured and predicted metabolic equivalent (*METs*) during resting (defined as the resting metabolic rate, *RMR*, <2 *METS*) and during light (2.0–3.9 *METS*) and moderate (4.0–5.9 *METS*) activities of daily living (*ADL*).

The ability of the random forest algorithm in estimate different levels of metabolic equivalent at rest (resting metabolic rate, *RMR*) and during light and moderate *ADL* is depicted in Fig. 4. These data were based on the same data displayed in Fig. 3A but clustered into groups according to the metabolic demand during *ADL*^29^. Less than 2% of the experimental data were composed by intense activities (>6 *METS*), therefore these data were excluded *a priori*. The proximity of the estimated *METS* to the equality line demonstrates that the random forest was able to dissociate between different metabolic demands. The proposed algorithm can be used to classify activity levels between light and moderate *ADL*.

### Aerobic system temporal dynamics

**Figure 5:**
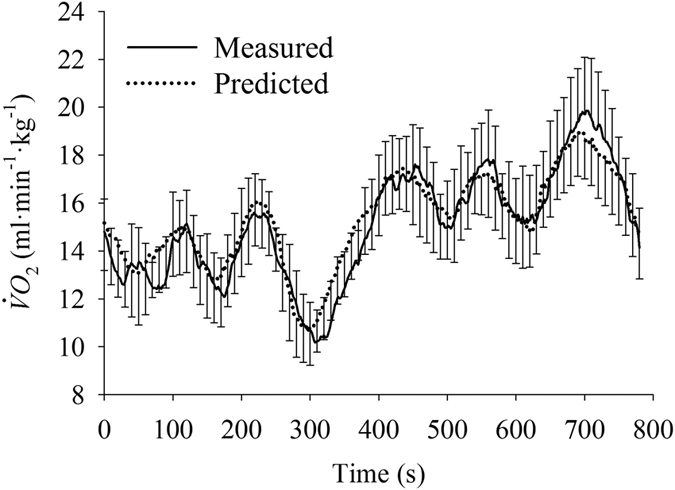
Second-by-second mean (lines, *n* = 16 per point) of the measured and predicted oxygen uptake (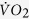) during pseudorandom ternary sequence over-ground walking protocol. The SD (upward vertical bars for measured values, downward for predicted) are plotted at 10 s intervals.

The group mean response for the second-by-second average 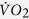 during the PRTS protocol was computed and depicted in Fig. 5.

**Figure 6:**
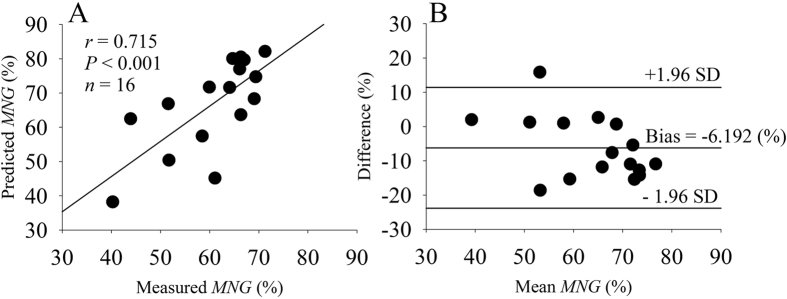
(**A**) linear correlation between the mean normalized gain (*MNG*) calculated from predicted and measured oxygen uptake data. (**B**) Bland-Altman plot of the data displayed in (**A**).

The comparison of the aerobic system temporal dynamics assessed by the *MNG* calculated from measured and predicted 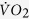 data during the PRTS protocol (Fig. 5) is displayed in Fig. 6. The *MNG* calculated from predicted 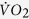 data was statistically similar (*P* = 0.136) and strongly, positively correlated to the *MNG* calculated from measured 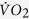 data. The *MNG* calculated from predicted 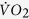 data presented a bias of −6.19% which corresponds to 10% of the average *MNG* response. The bias was statistically (*P* = 0.012) lower than the equality line (bias = 0). The 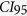 was 17.63% around the bias (or 29% of the mean *MNG* response).

## Discussion

In agreement to the initial hypothesis, the signals obtained from the wearable sensors allowed the prediction of oxygen uptake during activities of daily living and random paced walking. Aerobic system temporal dynamics assessed by the *MNG* from the predicted oxygen uptake were similar to those of oxygen uptake measured by a portable metabolic device. In addition, the random forest algorithm was able to identify physical activity levels and the resting metabolic demand.

Estimating the correct measurement of physical activity level during realistic scenarios remains a challenge^30^ and hence, new wearable technologies and data processing approaches are necessary. The quantification of the physical activity level usually involves the estimation of energy expenditure by indirect calorimetry. Indirect calorimetry has also been is used to calibrate wearable sensors for a wide range of activities during steady-state, allowing the energy expenditure estimation (i.e., steady-state 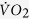) without the need for the original calorimetry measurement^31^. However, the complexity and diversity of *ADL* represent a challenge for the precise physical activity estimation in a realistic scenario^9^,^32^,^33^,^34^,^35^.

During randomly varying exercise intensities, assessment of the rate at which 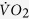 adapts to the metabolic demands is indicative of aerobic fitness^24^,^25^,^36^. Thus, the ability to predict 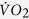 with an adequate time resolution provides an opportunity to obtain valuable information about cardiovascular health in addition to standard estimates of energy expenditure. Previous approaches to this problem have been restricted to studies conducted under controlled laboratory conditions^10^,^12^,^37^. In the present study, we investigated a simulated *ADL* protocol as well as an over-ground walking protocol (PRTS) that mimicked the dynamic changes in walking cadences expected during daily activities. The PRTS protocol offered an optimized stimulus for the aerobic system analysis through the study of the 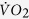 temporal dynamics and its prediction by a random forest machine learning regression model.

Recently, Altini *et al*.^9^ used a novel approach for estimating 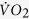 during nonsteady-state phases. Their algorithm combined an activity classification method with a numerical prediction approach that predicted 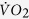 during dynamic phases of moderate *ADL*. However, the ability of the algorithm to correctly identify the 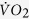 dynamics was reported only as a lower error of the estimation during exercise transitions. No further validation of the modelling parameters was carried out to explore the characterization of the aerobic adjustment dynamics with eventual health-related outcome.

In addition to *HR* and accelerometer^9^,^10^, the acquisition of more biological data such as 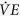 and *BF* improved the 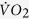 estimation during transitions and steady-state. When the *MNG* was calculated based on the predicted 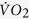 without considering 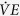 and *BF* as inputs, the *MNG* accuracy decreased by 55% (based on *r* value). Therefore, the integration of respiratory measurements for 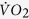 prediction seems to be indicated, evidencing some advantages of the smart-shirts over simpler wearable devices. As with the majority of the biological processes, 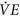 and *BF* signals are also delayed during transitions and despite not having exactly the same dynamics as the 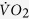, they have predictable relationships^38^ which would contribute to a better understanding of the biological variability during transitions.

Studies that optimize the 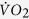 prediction during exercise transition with the intention to better estimate energy expenditure might be controversial. The *O*_2_ deficit at the on-transition phase is counter-balanced by the excess of *O*_2_ consumption during recovery^39^ thus the calorie counts based on different predicted 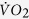 temporal dynamics should be almost similar. The energy expenditure estimation is independent on the 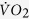 temporal dynamics, being determined only by the correct system static gain estimation. Therefore, in terms of calories (i.e., energy expenditure) calculated after a period of time, algorithms that successfully predict steady-state 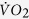 might be enough to estimate energy expenditure and no further methods are necessary for the 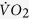 prediction during nonsteady-state phases. The justification for the correct 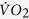 estimation during exercise transition has to have a reason beyond a “better” physical activity level estimation as considered next.

The 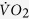 responses during transitions have been used to assess aerobic fitness in constrained settings^25^,^40^ and the expansion of these approaches outside of the laboratory environment represents the possibility to track changes in aerobic fitness and physical health on a daily basis. The assessment of aerobic fitness by wearable sensors during unsupervised daily living routine seems very promising. As demonstrated in Fig. 6, our algorithm was able to characterize the temporal dynamics (*MNG*) of the aerobic system based on the predicted 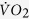 data. Therefore, the proposed algorithm can be used in the future for aerobic fitness assessment based on predicted 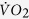 data obtained from wearable sensors during transitions encountered during *ADL* for ordinary people or patient populations, or during prescribed variations in work rate, such as athletic training.

## Study limitations

The purpose of the current study was to predict 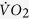 during the most common *ADL*. Thus, the exercise protocols were limited to light and moderate activities with intensities lower than ~6 *METs*, and any attempt to extend this range should include extensive testing for reliability. Any studies that investigate the algorithm proposed in the current study for high intensity activities must recognize that 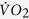 dynamics become more complex under these conditions with the potential for nonlinear contributions. The 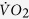 predictor developed in this study can be applied to evaluate the aerobic system dynamics during *ADL* where intense activities are unlikely to occur^28^.

The population tested in the current study (healthy men) had narrow weight and age ranges which might also restrict the use of the proposed algorithm. Further studies are necessary to verify the reliability of the 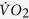 predictions in different populations. It is recommended that any future study incorporate dynamic protocols (such as the PRTS) to evaluate the ability of the proposed algorithms to predict the 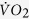 dynamics during exercise transitions.

## Conclusion

In conclusion, oxygen consumption dynamics can be predicted from the fusion of data from non-intrusive wearable sensors and machine learning prediction algorithms. Longitudinal predictions of oxygen uptake can be obtained from wearables based on the validation completed in the current study for activities of daily living and random over-ground walking. The proposed random forest ensemble predictor in conjunction with *MNG* can be used to investigate aerobic response during realistic activities with direct applicability for the general population. Developing the aforementioned predictive model will provide a unique opportunity for continued lifelong 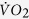 collections in unsupervised environments. This new technology provides a significant advance in ambulatory and continuous assessment of energy expenditure and aerobic fitness with potential for future applications such as the early detection of deterioration of physical health.

## Additional Information

**How to cite this article**: Beltrame, T. *et al*. Prediction of oxygen uptake dynamics by machine learning analysis of wearable sensors during activities of daily living. *Sci. Rep.* **7**, 45738; doi: 10.1038/srep45738 (2017).

**Publisher's note:** Springer Nature remains neutral with regard to jurisdictional claims in published maps and institutional affiliations.

## Supplementary Material

Supplementary Material

Supplementary Dataset 1
