# Supplementary material for: Prediction of oxygen uptake dynamics by machine learning analysis of wearable sensors during activities of daily living
Source: Sci Rep. 2017 Apr 5;7:45738. doi: 10.1038/srep45738 (PMC5381118; doi:10.1038/srep45738)
Supplement: Supplementary Material [file srep45738-s1.pdf]

Supplementary Material

Prediction of oxygen uptake dynamics by machine learning analysis of wearable sensors during activities of daily living

Beltrame T, Amelard R, Wong A, Hughson RL.

The following Matlab code (Code I) was used with wearable sensor data (Dataset I) in order to train the random forest machine learning algorithm and predict $\dot{V}O_2$. The leave-one-participant-out cross-validation was used to validate the algorithm. In addition, the mean of the normalized gains (MNG) calculation is described in this algorithm. As described at the end of Code I, sixteen random forests were generated from the leave-one-participant-out cross-validation. Based on the ensemble average of these random forests, a single final algorithm can be obtained.

Code I

```
% Beginning of the code
% LOAD_PHYSIOL_DATA Loads study physiology data.
%   [pidx,Mfeat] = LOAD_PHYSIOL_DATA loads the physiological data as
%   feature values (Mfeat) for each participant index (pidx). The "i"th row
%   of Mfeat is as feature vector for participant pidx(i).
%
%   [pidx,Mfeat,vo2] = LOAD_PHYSIOL_DATA loads the ground-truth measured
%   VO2 [mL/kg]
%
%   [pidx,Mfeat,vo2,Mdemog] = LOAD_PHYSIOL_DATA loads the participant
%   demographics (3 columns: weight, height, age)
```

```
%
%   [pidx,Mfeat,vo2,Mdemog,t2] = LOAD_PHYSIOL_DATA loads the times of the
%   pseudorandom protocol (t2).
function [pidx,Mfeat,vo2,Mdemog,t2] = load_physiol_data(filename)

% Read and pre-process csv file
filename = 'TB-File-01.csv';

% 1   2       3      4   5   6   7   8   9   10
% ID Weight   Height Age      BF       Ve        ACC     HR       CAD    VO2
data = csvread(filename);
data(any(data==-1,2),:) = [];
pidx = data(:,1);
vo2 = data(:,10)./data(:,2);
participants = unique(data(:,1));

% Extract feature vectors for each participant at every time step.
Mfeat = [data(:,[5,6,7,8,9]) zeros(size(data,1),1)];
for p = 1:numel(participants)
  Mfeat(pidx==participants(p),end) = [0; diff(Mfeat(pidx==participants(p),4))];
end

% "Pseudorandom protocols" last for 780s at beginning and end of trials.
% Time for last "pseudorandom protocol" (from timesync.xlsx)
participant_t2 = [2853, 2695,3369,3007,2753,2472,2500,3050,2840,2891,2941,2428,2965,2785,2714,2892];

% High pass filter
filtfreq = [0.01, Inf];  % Hz
for p = 1:numel(participants)
```

```
  pdata = Mfeat(pidx==participants(p),:);
  data_ts = timeseries(pdata,1:size(pdata,1));
  mu = mean(data_ts);
  data_filt = idealfilter(data_ts,filtfreq,'bandpass');
  Mfeat(pidx==participants(p),:) = bsxfun(@plus, data_filt.Data, mu);
end

%% Train random forest (leave one out cross validation)
fileout = 'y_yhat.csv';
if exist(fileout)
  delete(fileout)
end
Bfile = 'B.mat';

% Load training data
[pidx,Mfeat,vo2] = load_physiol_data('TB-File-01.csv');
participants = unique(pidx);

% Random forest parameters. Empirically determined.
minleafsize = 1;
ntrees = 9;

% Cell array of trees trained for each validation fold.
B = cell(1,numel(participants));

rmse = zeros(numel(participants),1);
rho = zeros(numel(participants),1);
% Train/test for each validation fold, and save random forest in B.
disp('Performing cross-validation validation...')
for p = 1:numel(participants)
```

```
  % Get training set participant ids
  trainidx = (pidx~=participants(p));
  % Get training and testing features and VO2
  train_x = Mfeat(trainidx,:);
  test_x = Mfeat(~trainidx,:);
  train_y = vo2(trainidx);
  test_y = vo2(~trainidx);

%   % Fix random seed for reproducability
%   rng(1)
    B{p} = TreeBagger(ntrees, train_x, train_y, ...
      'Method', 'regression', 'MinLeafSize', minleafsize);
    err_train = sum(B{p}.error(train_x,train_y,'mode','ensemble'));
    err_test = sum(B{p}.error(test_x,test_y,'mode','ensemble'));

  % Prediction plot
  test_yhat = predict(B{p},test_x);
  rmse(p) = sqrt(mean((test_yhat-test_y).^2));
  rho(p) = corr(test_yhat,test_y);

  % Write the actual and predicted VO2.
  dlmwrite(fileout, [test_y(:)'; test_yhat(:)'],'-append', ...
    'delimiter',',');
end
disp('Done')
save(Bfile,'B')
disp(sprintf('Saving file %s', Bfile))

%% MNG test (FFT analysis)
filein = 'y_yhat.csv';
```

```
disp(sprintf('Reading %s', filein))
fileout_fft = 'y_yhat_fft.csv';

[pidx,Mfeat,~,~,participant_t2] = load_physiol_data('TB-File-01.csv');
participants = unique(pidx);

% Goal: show pseudorandom protocol has spikes in frequency domain <0.01Hz
% Store accelerometer, vo2, and vo2hat data for processing
acc = Mfeat(:,3);
M = csvread(filein);
vo2 = M(1:2:end,:);
vo2est = M(2:2:end,:);

acc2 = zeros(size(vo2));
for i = 1:numel(participants)
  acc_pi = acc(pidx==participants(i));
  acc2(i, 1:numel(acc_pi)) = acc_pi;
end
acc = acc2;

% 300s warmup, t2 is the start of the 2nd pseudorandom protocol
vo2_protocol = zeros(numel(participants), 780);
vo2est_protocol = zeros(numel(participants), 780);
acc_protocol = zeros(numel(participants), 780);
for i = 1:numel(participants)
  t2 = participant_t2(i) + 300;
  vo2_protocol(i,:) = (vo2(i,300:300+779) + vo2(i,t2:t2+779))./2;
  vo2est_protocol(i,:) = (vo2est(i,300:300+779) + vo2est(i,t2:t2+779))./2;
  acc_protocol(i,:) = (acc(i,300:300+779) + acc(i,t2:t2+779))./2;
end
```

```
Fvo2_protocol = abs(fft(vo2_protocol,[],2))*2/780;
Fvo2est_protocol = abs(fft(vo2est_protocol,[],2))*2/780;
Facc_protocol = abs(fft(acc_protocol,[],2))*2/780;
figure,
subplot(3,1,1), plot(2:10,Facc_protocol(:,2:10)'), xlim([2 10]), title('acc')
subplot(3,1,2), plot(2:10,Fvo2_protocol(:,2:10)'), xlim([2 10]), title('vo2')
subplot(3,1,3), plot(2:10,Fvo2est_protocol(:,2:10)'), xlim([2 10]), title('vo2est')

vo2_freqs = Fvo2_protocol(:,[2,4,6,8]);
vo2est_freqs = Fvo2est_protocol(:,[2,4,6,8]);
acc_freqs = Facc_protocol(:,[2,4,6,8]);

% Compute system gain and normalize
vo2_gain = vo2_freqs./acc_freqs;
vo2_gain = bsxfun(@rdivide, vo2_gain, vo2_gain(:,1))*100;
vo2est_gain = vo2est_freqs./acc_freqs;
vo2est_gain = bsxfun(@rdivide, vo2est_gain, vo2est_gain(:,1))*100;
figure,
subplot(2,1,1), plot(vo2_gain','-x'), title('vo2 gain')
subplot(2,1,2), plot(vo2est_gain','-x'), title('vo2est gain')

vo2_mng = mean(vo2_gain(:,2:end),2);
vo2est_mng = mean(vo2est_gain(:,2:end),2);

[rho,pval] = corr(vo2_mng,vo2est_mng);
disp(sprintf('MNG R=%0.3g (p=%0.3g)',rho,pval))

delete(fileout_fft)
headers = ...
```

```
  {'ACC1','ACC3','ACC5','ACC7','VO2_1','VO2_3','VO2_5','VO2_7', ...
   'VO2est_1','VO2est_3','VO2est_5','VO2est_7','VO2_MNG','VO2est_MNG',...
   'rho'};
data = [acc_freqs vo2_freqs vo2est_freqs vo2_mng vo2est_mng ones(size(vo2_mng,1),1)*rho];
csvwrite_with_headers(fileout_fft,data,headers);

disp(sprintf('Saving file %s', fileout_fft))



%% Random jungle prediction
filein = 'Raw-04_dHR.csv';
disp(sprintf('Reading %s', filein))
fileout = 'Raw-04_VO2.csv';
delete(fileout)

[pidx,Mfeat,~,Mdemog] = load_physiol_data(filein);
ndata = size(Mfeat,1);
participants = unique(Mfeat(:,1));
% Predicted vo2 (ml/min)
vo2_est = zeros(ndata,1);

nforests = numel(B);
test_x = Mfeat(:,:);
pweight = Mdemog(:,1);
test_yhat = zeros(ndata,nforests);
disp('Predicting with random jungle...')
for p = 1:nforests
  test_yhat(:,p) = predict(B{p},test_x);
end
disp('Done')
```

```
% Random jungle (mean of individual trees)
mean_yhat = mean(test_yhat,2);
vo2_est = pweight.*mean_yhat;

csvwrite(fileout, [Mfeat vo2_est])
% End of code
```
